# A Rare Case Report of Amlodipine-Induced Gingival Enlargement and Review of Its Pathogenesis

**DOI:** 10.1155/2013/138248

**Published:** 2013-08-06

**Authors:** Sanjeev Joshi, Sucheta Bansal

**Affiliations:** ^1^Department of Prosthodontics Including Crown and Bridge & Implantology, Himachal Institute of Dental Sciences, Paonta Sahib, Himachal Pradesh 173025, India; ^2^Department of Oral and Maxillofacial Pathology, Himachal Institute of Dental Sciences, Paonta Sahib, Himachal Pradesh 173025, India

## Abstract

Gingival enlargement is a common clinical feature of gingival and periodontal diseases. It is an unwanted side effect of certain systemic drugs given for nondental treatment. It is being reported with three main groups of drugs like calcium channel blockers (CCBs), immunosuppressants, and anticonvulsants. Among calcium channel blockers, nifedipine causes gingival hyperplasia in about 10% of patients, whereas the incidence of amlodipine-, a third generation calcium channel blocker, induced gingival hyperplasia is very limited. There are very few reports of amlodipine-induced gingival enlargement at a dose of 5 mg. We report a case of amlodipine-induced gingival enlargement in a 45-year-old hypertensive patient taking amlodipine at a dose of 5 mg.

## 1. Introduction

Drug-induced gingival enlargement was first reported in 1939 by Kimball with chronic usage of the antiepileptic drug phenytoin [1]. Currently, more than 20 prescription medications are associated with gingival enlargement [2]. Drugs associated with gingival overgrowth can be broadly categorized into three major groups according to their therapeutic actions, namely, anticonvulsants, immunosuppressants, and calcium channel blockers [3, 4].

Amlodipine is a new dihydropyridine calcium channel blocker that is used in the management of both hypertension and angina. Ellis et al. [5] first reported gingival sequestration of amlodipine and amlodipine-induced gingival overgrowth. Since then, very few cases of amlodipine-induced gingival hyperplasia have been reported in the dental literature although there are numerous reports of nifedipine- (another member of calcium channel blockers) induced gingival overgrowth till date. There are less data on reports of hyperplasia with amlodipine at a dose of 5 mg, even after taking it for more than 6 months [6, 7]. 

But, in the present case, the gingival hyperplasia occurred at a dose of 5 mg within 6 months of use.

## 2. Case Report

A 45-year-old male patient came to the department with the chief complaint of loose teeth in upper and lower front jaw regions since 1 year with swollen and bleeding gums. Patient first noted bead like nodular growth over the gums which progressively enlarged to the present size covering almost entire teeth interfering with further cleaning of teeth.

The patient was hypertensive since 1.5 years and was under medication Coronol-AM (atenolol, 50 mg + amlodipine, 5 mg) once daily. He denied the history of any adverse habits. 

The patient was moderately built and nourished with no signs of anaemia and jaundice and noncyanosed. His vital signs were within the normal range. 

**Figure 1:**
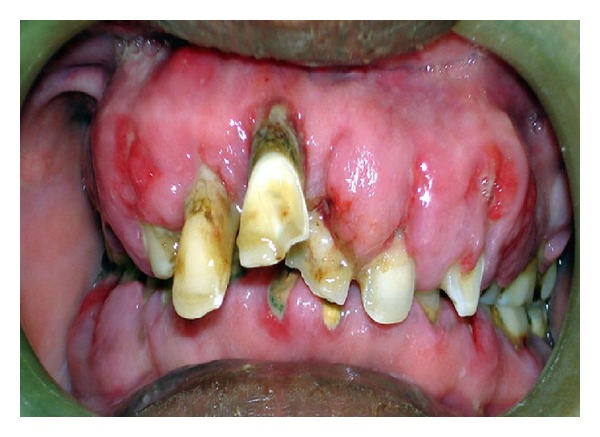
Showing generalized amlodipine-induced gingival enlargement.

Intraoral examination revealed generalized enlargement of attached gingival extending up to marginal and interdental gingiva. Surface of the gingiva appears lobulated with loss of scalloping (Figure 1). Poor oral hygiene status of patient was assessed by the presence of local irritating factors which surrounded the teeth. 

**Figure 2:**
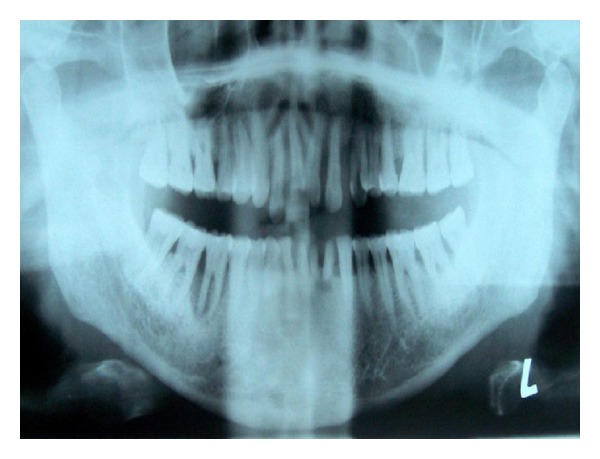
Orthopantomography revealed generalized bone loss.

Based on drug history and clinical examination of the patient provisional diagnosis of combined gingival enlargement was made. Complete hemogram of the patient was done, but all the parameters were within the normal range. Orthopantomogram was taken which revealed generalized bone loss (Figure 2).

After this, incisional biopsy was done. Histopathological report revealed few areas of hyperplastic orthokeratinized and parakeratinised stratified squamous epithelium and connective tissue exhibiting mixture of dense and loose fibrous component. Inflammatory cell infiltrate with PMLs and dilated blood capillaries with few areas of calcifications were also evident.

**Figure 3:**
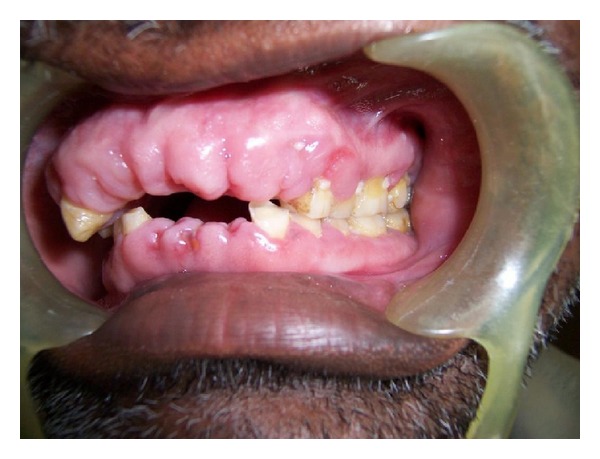
Showing response to therapy after 1.5 months.

Correlating history, clinical examination, and investigations, final diagnosis of combined gingival enlargement (amlodipine induced and inflammatory) was made. Patient was referred to periodontics department for further treatment. In the preliminary phase, extraction of teeth (11, 21, 22, 23, 31, 32, and 34) with hopeless prognosis was recommended. Planned sessions of scaling and root planning with drug change with the patient's physician consent were performed. Patient was put on tablet Normadate 100 mg twice daily and was evaluated after the period of 1.5 months. There was drastic change in the clinical picture of gingiva with complete loss of inflammatory component (Figure 3).

## 3. Discussion

Amlodipine is a 3rd generation dihydropyridine calcium antagonist which is structurally similar to nifedipine but pharmacodynamically comparable to it. In patients with hypertensive heart disease the prevalence of gingival overgrowth associated with amlodipine is lower than that associated with other calcium channel blocking agents including nifedipine [6]. Drug-induced gingival overgrowth usually occurs within the first 3 months of starting drug therapy at a dose of 10 mg/day and begins as an enlargement of the interdental papilla. Although few cases of amlodipine-induced hyperplasia have been reported, the present case is interesting as it occurred with a low dose of amlodipine (5 mg) and appeared on administration for 6 months.

Seymour et al. [8] gave a review on the pathogenesis of drug-induced gingival overgrowth in which they considered it as a multifactorial model, involving an interaction of several factors, which expands on the interaction between drug and metabolite with the gingival fibroblasts. Predisposing factors for these changes are age, genetic predisposition, pharmacokinetic variables, drug-induced alterations in gingival connective tissue homeostasis, histopathology, ultrastructural factors and inflammatory changes, and drug-induced action on growth factors.

The underlying mechanism behind drug-induced gingival hyperplasia involves inflammatory and noninflammatory pathways. The proposed noninflammatory mechanisms include defective collagenase activity due to decreased uptake of folic acid, blockage of aldosterone synthesis in adrenal cortex, and consequent feedback increase in adrenocorticotropic hormone level and upregulation of keratinocyte growth factor. Alternatively, inflammation may develop as a result of direct toxic effects of concentrated drug in crevicular gingival fluid and/or bacterial plagues. This inflammation could lead to the upregulation of several cytokine factors such as transforming growth factor-*β*1 [9–11].

Many studies have been conducted which showed that amlodipine cannot induce gingival hyperplasia at 5 mg once daily dose even if taken for more than 6 months. It can be caused only at a dose of 10 mg/day [6, 8]. The present case is unique in that even 5 mg/day dose of amlodipine caused gingival hyperplasia after 6 months of use.

The mechanism through which these drugs induce gingival enlargement is still poorly understood. It has been found that phenytoin and calcium channel blockers inhibit the intracellular Ca^2+^ uptake thereby stimulating gingival fibroblasts. Not all the patients receiving the same drug develop gingival enlargement. Possible reason can be that individuals with gingival enlargement have fibroblasts with an abnormal susceptibility to the drug. It has also been proposed that the susceptibility to pharmacologically induced gingival enlargement may be governed by existence of differential proportions of fibroblast subset in each individual which exhibit a fibrogenic response to these medications. It has also been shown that the functional heterogenicity exists in gingival fibroblasts in response to various stimuli [12]. 

A synergestic enhancement of collagenous protein synthesis by human gingival fibroblasts is found when these cells are exposed simultaneously to calcium channel blockers and elevated levels of interleukin-1*β* (a proinflammatory cytokine) in inflamed gingival tissues. Interleukin-6 also plays a role in fibrogenic responses of gingiva to these medications. Interleukin-6 targets fibroblasts which trigger the proliferation of fibroblasts and exert the positive regulation on collagen and glycosaminoglycans synthesis. So this cytokine has been proposed to play a pathogenic role in fibrotic gingival enlargement [8].

 Clinical relevant doses of cyclosporins trigger gingival fibroblasts to exhibit significant reduced levels of matrix metalloproteinases-1 and -3 secretions which lead to accumulation of extracellular matrix components [13]. There is a strong correlation between the production of inactive collagenase and responding fibroblasts. Because of reduced folic acid uptake, there is limited production of activator protein which converts inactive collagenase to active collagenase. Limited amount of collagenase becomes available [8].

 Treatment consists of stopping the offending drug if possible with the patient's physician consent and providing the supplements of folic acid and ascorbic acid. Reduction in the size of the gingival overgrowth has been reported within a week of drug withdrawal and may lead to full resolution [14]. 

Patients benefit from effective oral hygiene measures, professional tooth cleaning, scaling, and root planning [15]. If gingival enlargement persists after careful consideration of the previously mentioned approaches, these cases need to be treated by surgery, either by gingivectomy or flap surgery. In present case patient was subjected to planned sessions of scaling and root planning with substitute drug Normadate 100 mg twice daily. On evaluating the patient after the period of 1.5 months drastic change in the clinical picture of gingiva with complete loss of inflammatory component was seen.

## 4. Conclusion

We conclude that the gingival hyperplasia could occur with amlodipine even at a small dose (5 mg). Physicians and dentists should be aware of the etiologic medications that can induce gingival hyperplasia and be able to identify changes in the oral cavity in such patients and to prevent, diagnose, and successfully manage them. It can be treated locally and systemically with combined effort of medical and dental physician. So, cooperative teamwork between the patient, his physician, and the dental health care professional is mandatory to minimize and successfully treat such unwanted side effects of drugs.
